# Serum metabolic biomarkers for synucleinopathy conversion in isolated REM sleep behavior disorder

**DOI:** 10.1038/s41531-021-00184-9

**Published:** 2021-05-13

**Authors:** Ariadna Laguna, Helena Xicoy, Eduardo Tolosa, Mònica Serradell, Dolores Vilas, Carles Gaig, Manel Fernández, Oscar Yanes, Joan Santamaria, Núria Amigó, Alex Iranzo, Miquel Vila

**Affiliations:** 1 Neurodegenerative Diseases Research Group, Vall d’Hebron Research Institute (VHIR)—Network Center for Biomedical Research in Neurodegenerative Diseases (CIBERNED), Barcelona, Spain; 2 Department of Cell Biology, Radboud Institute for Molecular Life Sciences, Radboud University Medical Centre, Nijmegen, The Netherlands; 3 Department of Molecular Animal Physiology, Donders Institute for Brain, Cognition and Behaviour, Donders Centre for Neuroscience, Faculty of Science, Nijmegen, The Netherlands; 4 Parkinson Disease and Movement Disorders Unit, Neurology Service, Hospital Clinic de Barcelona, Universitat de Barcelona IDIBAPS, CIBERNED: CB06/05/0018-ISCIII, Barcelona, Spain; 5 Center for Sleep Disorders, Neurology Service, Hospital Clinic Barcelona, Universitat de Barcelona, IDIBAPS, CIBERNED: CB06/05/0018-ISCIII, Barcelona, Spain; 6 Metabolomics Platform, IISPV, Department of Electronic Engineering, Universitat Rovira i Virgili—Spanish Biomedical Research Center in Diabetes and Associated Metabolic Disorders (CIBERDEM), Tarragona, Spain; 7 Biosfer Teslab, Reus, Spain; 8 Department of Biochemistry and Molecular Biology, Autonomous University of Barcelona, Barcelona, Spain; 9 Catalan Institution for Research and Advanced Studies (ICREA), Barcelona, Spain

**Keywords:** Predictive markers, Neurodegeneration, Parkinson's disease

## Abstract

Isolated rapid eye movement (REM) sleep behavior disorder (iRBD) is a prodromal stage of Lewy-type synucleinopathies (LTS), which can present either with an initial predominant parkinsonism (Parkinson’s disease (PD)) or dementia (dementia with Lewy bodies (DLB)). To provide insights into the underlying pathogenic mechanisms, the lipoprotein and protein glycosylation profile of 82 iRBD patients, collected before and/or after their conversion to an overt LTS, and 29 matched control serum samples were assessed by nuclear magnetic resonance (NMR) spectroscopy. Data were statistically analyzed to identify altered metabolites and construct predictive models. Univariant analysis detected no differences between iRBD patients with an LTS compared to controls. However, significant differences were found when the analysis distinguished between iRBD patients that manifested initially predominant parkinsonism (pre-PD) or dementia (pre-DLB). Significant differences were also found in the analysis of paired iRBD samples pre- and post-LTS diagnosis. Predictive models were built and distinguished between controls and pre-DLB patients, and between pre-DLB and pre-PD patients. This allowed a prediction of the possible future clinical outcome of iRBD patients. We provide evidence of altered lipoprotein and glycosylation profiles in subgroups of iRBD patients. Our results indicate that metabolic alterations and inflammation are involved in iRBD pathophysiology, and suggest biological differences underlying the progression of LTS in iRBD patients. Our data also indicate that profiling of serum samples by NMR may be a useful tool for identifying short-term high-risk iRBD patients for conversion to parkinsonism or dementia.

## Introduction

Neurodegeneration associated with aggregated α-synuclein in the form of Lewy bodies and neurites (Lewy-type synucleinopathy; LTS) manifests clinically as a parkinsonism, usually associated with late onset dementia (i.e., Parkinson’s disease (PD))^1^, although dementia can also appear as an early feature and is then designated as dementia with Lewy bodies (DLB)^2^. The biological basis underlying this clinical heterogeneity remains poorly understood.

Isolated rapid eye movement (REM) sleep behavior disorder (iRBD) is a parasomnia characterized by vigorous dream-enacting behaviors and loss of REM sleep muscle atonia^3,4^. Although iRBD patients have no overt neurological diseases, long-term prospective studies show that over 90% of them develop an LTS within 14 years after iRBD diagnosis, and the remaining disease-free patients show short-term risk markers for developing an LTS^5,6^. As such, iRBD is a clinical marker of the prodromal stage of LTS^7^, and provides an excellent opportunity to study LTS at early stages. This is important for management at clinical onset, development of early disease-modifying interventions, and selection and stratification in future neuroprotective trials.

iRBD and LTS have been associated with cardiovascular risk factors^8–11^ and inflammatory processes^12,13^. Nuclear magnetic resonance (NMR)-based tests are a robust method to characterize lipoproteins and protein glycosylation profiles in intact serum samples^14^, which have been used to study metabolic changes^15–18^ and systemic inflammatory processes^19,20^. However, this type of approach has not been explored before in the context of iRBD.

Herein we evaluated the lipoprotein and protein glycosylation profiles of serum samples from iRBD patients by NMR and assessed whether the baseline profile identifies the future development of an LTS, and whether that manifests initially with parkinsonism or dementia.

## Results

### Cohort characteristics

The total number of samples analyzed was 130, including 101 from iRBD patients (i.e., 33 from iRBD-only, 33 from pre-LTS, among which 15 were pre-DLB and 18 pre-PD, and 35 from post-LTS, among which 20 were post-DLB and 15 post-PD, individuals), and 29 from controls (Table 1). There were no differences between groups in gender, assessed with Chi-square with Yates’ correction, except for control vs. post-DLB samples (*p* value = 0.0373). There were also no differences in the age at iRBD nor LTS diagnosis, assessed by mean ranks comparison and corrected for multiple comparison. Moreover, there were no differences of time between iRBD and LTS diagnosis, sample collection and LTS diagnosis (pre-groups), and LTS diagnosis and sample collection (post-groups). There were only age differences at sample collection between controls and post-LTS (*p* value = 0.036), controls and post-DLB samples (*p* value = 0.0425), pre-LTS and post-LTS samples (*p* value = 0.0019), and pre-DLB and post-DLB samples (*p* value = 0.0198). Comparably, there were differences in the time between iRBD diagnosis and sample collection between pre-LTS and post-LTS (*p* value < 0.0001), and pre-DLB and post-DLB samples (*p* value = 0.0002). These differences are inherent to the timeline of iRBD and LTS onset. Similar results were obtained when considering only the paired samples from each group (Table 1).

**Table 1:** Demographic information on the serum samples analyzed.

	Control	iRBD-only	Pre-LTS	Pre-LTS	Pre-DLB	Pre-DLB	Pre-PD	Pre-PD	Post-LTS	Post-LTS	Post-DLB	Post-DLB	Post-PD	Post-PD
			All	Paired	All	Paired	All	Paired	All	Paired	All	Paired	All	Paired
Number of samples	29	33	33	19	15	12	18	7	35	19	20	12	15	7
Gender, male/female	19/10	28/5	25/8	15/4	14/1	11/1	11/7	4/3	31/4	15/4	**19/1**^a^	11/1	12/3	4/3
Age at iRBD diagnosis		69.2 ± 5.3	69.3 ± 5.2	69.6 ± 5.4	69.5 ± 5.7	69.4 ± 1.7	69.2 ± 4.8	70.1 ± 4.5	70.1 ± 4.9	69.6 ± 5.4	70.5 ± 5.6	69.4 ± 1.7	69.7 ± 4.0	70.1 ± 4.5
BMI at iRBD diagnosis	27.2 ± 6.4^b^	27.8 ± 3.6	27.8 ± 3.5	27.4 ± 3.1	26.9 ± 3.2	26.5 ± 3.4	28.1 ± 3.8	28.9 ± 1.8	26.6 ± 3.6	27.4 ± 3.1	25.8 ± 3.9	26.5 ± 3.4	27.7 ± 2.7	28.9 ± 1.8
Ever smokers at iRBD diagnosis, *n* (%)	(38)^b^	19 (63)	14 (52)	9 (64)	8 (67)	6 (67)	6 (40)	3 (60)	17 (74)	9 (64)	8 (67)	6 (67)	9 (82)	3 (60)
Dyslipidemia at iRBD diagnosis, *n* (%)	(38)^b^	10 (31)	10 (30)	4 (21)	4 (27)	2 (17)	6 (33)	2 (29)	4 (11)	4 (21)	2 (10)	2 (17)	2 (13)	2 (29)
Diabetes mellitus (type 2) at iRBD diagnosis, *n* (%)	(22)^b^	5 (16)	6 (18)	5 (26)	3 (20)	2 (17)	3 (17)	3 (43)	8 (23)	5 (26)	3 (15)	2 (17)	5 (33)	3 (43)
Arterial hypertension at iRBD diagnosis, *n* (%)	(48)^b^	14 (44)	9 (27)	5 (26)	3 (20)	1 (8)	6 (33)	4 (57)	12 (34)	5 (26)	4 (20)	1 (8)	8 (53)	4 (57)
Age at LTS diagnosis			76.8 ± 4.3	76.5 ± 4.3	76.3 ± 4.2	75.8 ± 4.3	77.1 ± 4.5	77.6 ± 4.3	75.2 ± 4.4	76.5 ± 4.3	75.7 ± 4.1	75.8 ± 4.3	74.5 ± 4.9	77.5 ± 4.3
Time LTS diagnosis from iRBD diagnosis			7.5 ± 3.5	6.8 ± 3.1	6.8 ± 3.1	6.4 ± 3.1	8.0 ± 3.7	7.5 ± 3.2	**5.1** ± **3.3**^**c**^	6.8 ± 3.1	5.3 ± 3.1	6.4 ± 3.1	4.9 ± 3.6	7.5 ± 3.2
Age at sample collection	72.6 ± 7.6	74.2 ± 5.2	71.8 ± 4.9	71.2 ± 5.4	71.2 ± 5.1	70.6 ± 5.4	72.3 ± 4.8	72.1 ± 5.7	**76.8** ± **4.5**^**a,c**^	**77.7** ± **4.3**^c^	**77.3** ± **4.2**^a,d^	77.2 ± 4.3	76.2 ± 5.0	78.4 ± 4.7
Time sample collection from iRBD diagnosis		4.9 ± 3.9	2.5 ± 3.2	1.5 ± 2.0	1.7 ± 2.3	1.2 ± 1.5	3.2 ± 3.7	2.1 ± 2.7	**6.7** ± **3.2**^c^	**8.0** ± **3.1**^c^	**6.8** ± **3.2**^d^	**7.9** ± **3.1**^d^	6.5 ± 3.4	**8.4** ± **3.2**^e^
Time LTS diagnosis from sample collection			4.7 ± 2.2	5.3 ± 2.5	5.1 ± 2.3	5.2 ± 2.5	4.3 ± 2.2	5.5 ± 2.7						
Time sample collection from LTS diagnosis									1.6 ± 1.7	1.2 ± 1.3	1.6 ± 1.7	1.4 ± 1.4	1.6 ± 1.8	0.9 ± 1.0
Time between paired samples				6.7 ± 2.5		6.8 ± 2.3		6.5 ± 3.0		6.7 ± 2.5		6.8 ± 2.3		6.5 ± 3.0

Data are included for the whole group, including both paired and unpaired samples (All), and only for paired samples (Paired). The table includes the number of samples, gender ratio (male/female), mean age at isolated rapid eye movement (REM) sleep disorder (iRBD) diagnosis (years), body max index (BMI) at iRBD diagnosis, ever smokers at iRBD diagnosis (number of positives (percentage of positives), *n* (%)), dyslipidemia at iRBD diagnosis (*n* (%)), diabetes mellitus at iRBD diagnosis (*n* (%)), arterial hypertension at iRBD diagnosis (*n* (%)), mean age at Lewy-type synucleinopathy (LTS) diagnosis (years), mean age at sample collection (years), time between iRBD diagnosis and sample donation (years), time between sample collection and LTS diagnosis (years), time between LTS diagnosis and sample collection (years), and time between paired samples (years). DLB, dementia with Lewy bodies; PD, Parkinson’s disease; Pre, samples obtained before the onset of the overt neurodegenerative disease; Post, samples obtained after the onset of the overt neurodegenerative disease. Gender, BMI, smoking habits, dyslipidemia, diabetes mellitus, and arterial hypertension differences were assessed by Chi-square with Yates’ correction. Age and time differences from the whole group (All) and the paired samples (Paired) were assessed by Wilcoxon signed-rank test or the paired and non-parametric test Wilcoxon matched-pairs signed-rank test, respectively, combined with the Benjamini–Hochberg procedure to correct for false discovery rate. Statistically significant differences are highlighted in bold.

^a^Significantly different from control.

^b^Data obtained from the health survey (Catalonia, Spain, 2018), considering individuals 65–74 years old.

^c^Significantly different from pre-LTS.

^d^Significantly different from pre-DLB.

^e^Significantly different from pre-PD.

We accounted for relevant comorbidities (i.e., obesity, tobacco use, dyslipidemia, diabetes mellitus type 2 and arterial hypertension; categorical data are given as percentage of total in Table 1) and no differences between iRBD groups were detected at the time of iRBD diagnosis, assessed with Chi-square with Yates’ correction. Similarly, no differences were found when comparing the prevalence of these comorbidities between our iRBD groups and the general population of individuals aged 64–75 years old in our region Catalonia^21^ (considered as controls) (Table 1).

### Differences between iRBD-LTS patients and controls

Twenty-seven parameters related to lipoproteins and protein glycosylation were analyzed in 130 serum samples, including controls, pre-LTS (which comprise pre-DLB and pre-PD), and post-LTS (which comprise post-DLB and post-PD) iRBD patients. No significant differences were found between pre-LTS or post-LTS iRBD patients compared with controls (Supplementary Table 1). However, when iRBD-LTS patients were analyzed distinguishing between those with either PD or DLB, we found significant decreased *area glycB* in pre-DLB patients (335 ± 67 µmol/L) compared to control subjects (431 ± 115 µmol/L), with a false discovery rate (FDR)-corrected *p* value of 0.006 (Supplementary Table 1).

### Differences between iRBD patients before and after LTS diagnosis

**Fig. 1:**
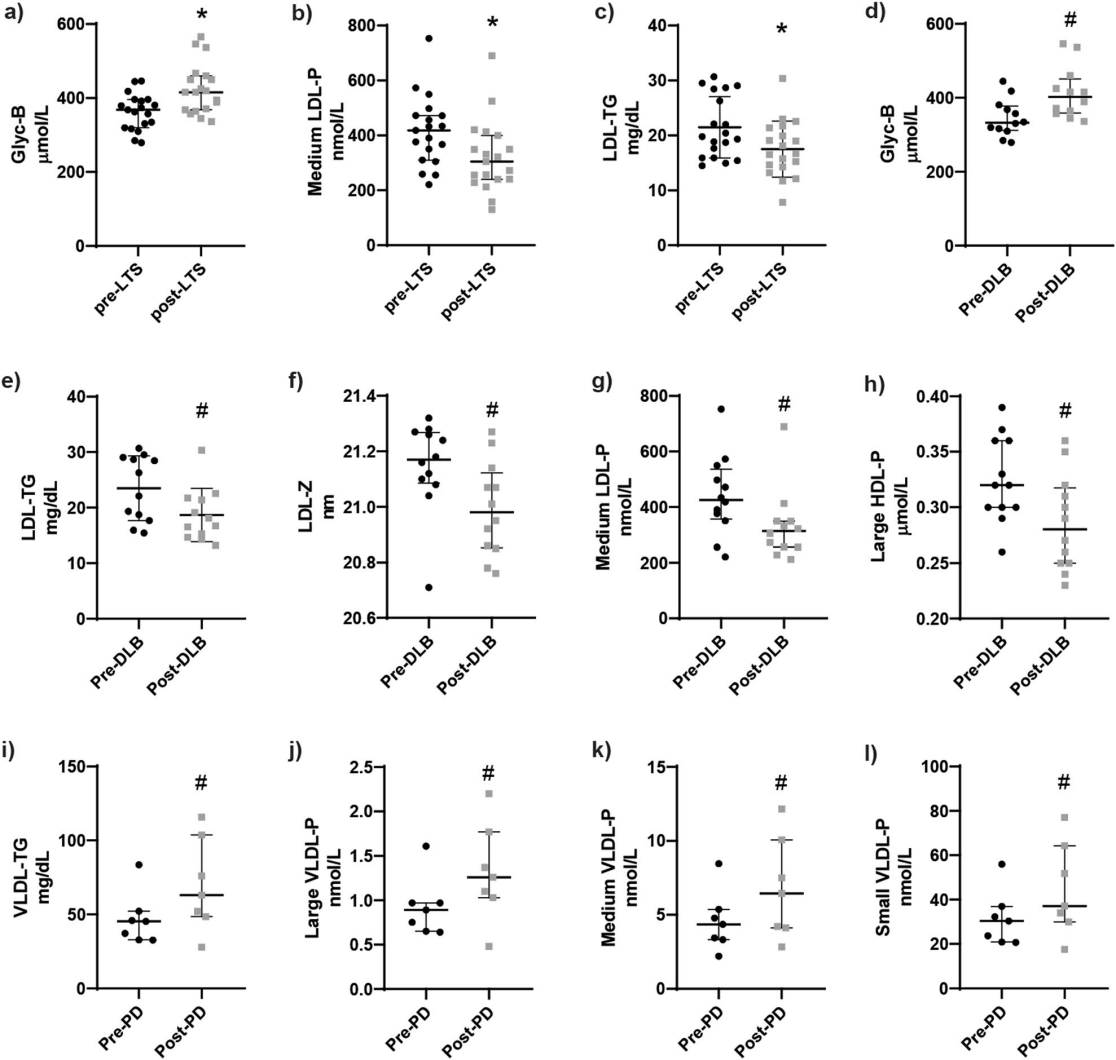
Differences between iRBD patients before and after LTS diagnosis. Graphs of the **a** concentration of glycosylated protein B (Glyc-B), **b** concentration of medium sized LDL particles (medium LDL-P), and **c** concentration of triglycerides in LDL (LDL-TG) in the same iRBD patients, before (pre-) and after (post-) the diagnosis of LTS. Graphs of the **d** concentration of Glyc-B, **e** concentration of LDL-TG, **f** size of LDL (LDL-Z), **g** concentration of medium LDL-P, and **h** concentration of large HDL particles (large HDL-P) in the same IRBD patients, before (pre-) and after (post-) the diagnosis of DLB. Graphs of the **i** concentration of triglycerides in VLDL (VLDL-TG), **j** large VLDL particles (large VLDL-P), **k** medium VLDL particles (medium VLDL-P), and **l** small VLDL particles (small VLDL-P) in the same IRBD patients, before (pre-) and after (post-) the diagnosis of PD. Mean with interquartile range represented. Wilcoxon matched-pairs signed-rank test, combined with the Benjamini–Hochberg procedure to correct for false discovery rate (FDR). *FDR-adjusted *p* value < 0.05. ^#^FDR-adjusted *p* value < 0.25.

The serum lipoprotein and glycosylated protein profiles from those 19 (12 DLB and 7 PD) iRBD patients for which we had paired samples, from before and after their diagnosis of LTS, were compared. Significantly (FDR-corrected *p* value < 0.05) higher levels of *area glycB*, and lower levels of *medium LDL-P* and *LDL-TG* were found in iRBD patients after the diagnosis of an LTS compared to the same patients before the diagnosis (Fig. 1a–c). When iRBD-LTS patients were analyzed distinguishing between those with either PD or DLB, we could not find significant differences, likely due to the small number of samples in each comparison. However, a trend (FDR-corrected *p* value < 0.25) towards lower levels of *LDL-TG*, *LDL-Z*, *medium LDL-P*, and *large HDL-P*, and higher levels of *area glycB* were found in iRBD patients after the diagnosis of DLB, compared to the same patients before the diagnosis (Fig. 1d–h). Moreover, a trend towards increased levels of serum *VLDL-TG*, and *large, medium*, and *small VLDL-P* was found in iRBD patients after the diagnosis of PD, compared to the same patients before the diagnosis (Fig. 1i–k).

### Predictive model building

**Fig. 2:**
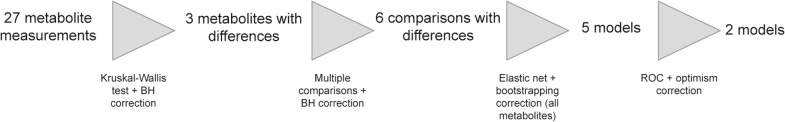
Schematic representation of model building workflow. Scheme illustrating the input/output of each step done for building models to discriminate between groups, and the statistical methods used to perform them. BH Benjamini–Hochberg, ROC receiver operating characteristics.

**Fig. 3:**
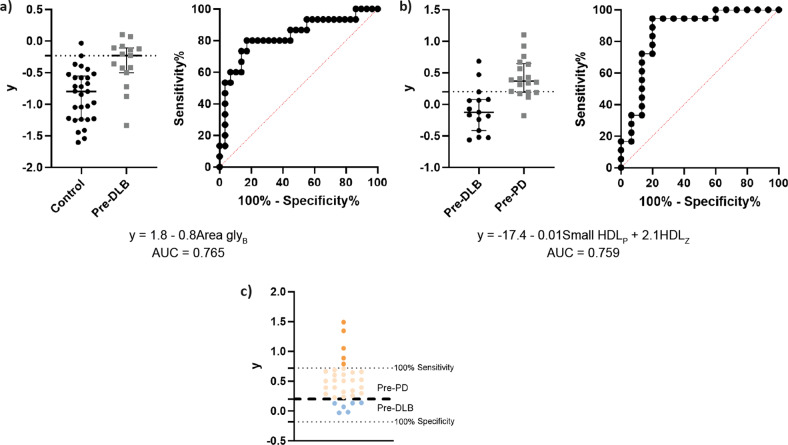
Discriminatory models. Graphs showing the results of the equation (written underneath) that allows distinguishing between controls and pre-DLB patients (**a**), and pre-DLB and pre-PD patients (**b**), including the threshold with the highest likelihood ratio (gray dotted line), together with the pertinent ROC curve and area under the curve (AUC) values. The application of model (**b**) in iRBD-only samples (**c**) distinguishes between putative pre-DLB (blue dots) and pre-PD (orange dots, darker orange implies 100% sensitivity) patients.

To identify biomarkers with a possible predictive value, a machine learning approach was used to build predictive models (Fig. 2). Models with a corrected area under the curve (AUC) > 0.75 were built for two different comparisons (control vs. pre-DLB and pre-DLB vs. pre-PD) with all variables. In agreement with our first analysis, an equation was obtained distinguishing between controls and pre-DLB patients that included *area glyc*B (Fig. 3a). This gave a model with a corrected AUC of 0.765 (*p* value <0.0004), a sensitivity of 53.33%, and a specificity of 96.55% at a cut-off of *y* > −0.233. Second, an equation was obtained distinguishing between pre-DLB and pre-PD patients that included small *HDL-P* and *HDL-Z* (Fig. 3b). This gave a model with a corrected AUC of 0.759 (*p* value < 0.0005), a sensitivity of 72.22%, and a specificity of 86.67% at a cut-off of *y* > 0.201. Finally, we applied the model that distinguished between pre-DLB and pre-PD patients to 33 iRBD patients that remained disease-free at the end of the study (iRBD-only, which had a mean follow-up between iRBD diagnosis and the time of this study of 10.3 ± 4.1 years, and between sample collection and the time of this study of 5.4 ± 1.7 years). This allowed the prediction that six individuals would classify as pre-DLB patients, while the rest would classify as pre-PD patients (Fig. 3c). Additionally, five of the putative pre-PD patients were classified as such with a sensitivity of 100% (Fig. 3c).

## Discussion

Here, we assessed serum lipoprotein and protein glycosylation profiles in iRBD patients before and after the manifestation of an overt LTS. We found that patients with iRBD present changes in their serum metabolic profile that differ from before and after their LTS diagnosis, and also importantly, that differ between those eventually developing predominant parkinsonism (pre-PD) or dementia (pre-DLB) at onset.

Currently, existing short-term conversion biomarkers in iRBD to LTS include hyposmia^22^, altered DAT-SPECT^23^, and pareidolias^24^, the latter being more associated to DLB. Also, few biochemical peripheral biomarkers have been suggested to identify iRBD from healthy controls, including impaired ghrelin excretion^25^, serum N-glycan composition^26^, and serum proteins, such as dopamine β-hydrolase^12^. Nevertheless, all these marker studies compared iRBD patients with controls, without considering that the patients might be a heterogeneous group including individuals close to phenoconversion to an LTS manifesting primarily with parkinsonism or dementia.

Here, using two different analysis methods, we observed that the parameter *area glycB* could be considered as a biomarker for iRBD patients that develop a dementia onset LTS. This measurement corresponds to the amount of *N*-acetylneuraminic acid in glycoproteins. *N*-acetylneuraminic acid, the most abundant form of sialic acid in human cells, has a negative charge and hydrophilicity, which enable its structural and modulatory roles, such as blood cell charge repulsion or neural plasticity^27^. Sialylation changes have been associated with infectious diseases, stress, inflammation, autoimmune diseases, cancer, and neurodegeneration^28^. As such, changes in *area glycB* have been described as an inflammatory marker associated with insulin resistance and adiposity^29^, obesity^20^, and rheumatoid arthritis^19^ (reviewed in ref. ^30^). In our data set, although there were no differences in the prevalence of cardiovascular risk factors (i.e., obesity, tobacco use, dyslipidemia, diabetes mellitus, and arterial hypertension), *area glycB* was decreased in pre-DLB patients compared to controls, but then there was a trend to increased *area glycB* in post-DLB compared to pre-DLB condition. This parameter is not modified in any of the analysis considering our PD cohort, implying possible biochemical differences in the patients manifesting these two different phenotypes. A few recent studies reported differences in peripheral cytokines between iRBD patients and healthy controls^31–33^, in particular, increased serum/plasma TNF-alpha levels in iRBD compared to controls. Furthermore, their data showed higher levels at baseline in those patients who later converted to LTS compared to those iRBD non-converters. Although there are no studies specifically on the inflammatory profile of iRBD patients before developing dementia onset LTS, peripheral inflammation has been seen in early stages of manifest DLB^34,35^. This phenomenon could be associated with the observed trend to increased inflammatory markers during the neurodegeneration that spans from iRBD to DLB conversion, although the post-DLB levels are like those in control individuals. Further studies are needed to elucidate the role of peripheral immune changes in the pathogenesis of neurodegeneration in the prodromal stage of LTS.

The analysis of samples from the same iRBD patients before and after their LTS diagnosis revealed higher levels of *area glycB*, and lower levels of *medium LDL-P* and *LDL-TG* after the diagnosis of an LTS. Higher levels of *area glycB* would point towards a more inflammatory profile when patients have an overt neurological condition, as also suggested by our previously discussed results. On the other hand, lower levels of *medium LDL-P* and *LDL-TG* would point towards a lower cardiovascular risk^36,37^, which is in disagreement with previous findings associating iRBD with higher cardiovascular risk factors^8^. It should be noted that these three parameters showed the same trend when analyzing the DLB, but not the PD group. Since DLB patients (*n* = 12) outnumbered the PD patients (*n* = 7) in the paired samples, it is possible that these changes are not common for both LTS manifestations, but rather specific for DLB. Studies with a larger cohort are needed to address these ambiguities and draw functional conclusions about the results.

We built a model to distinguish between iRBD patients that will eventually evolve into an LTS with an initial parkinsonism or dementia. The identification of a predictive biomarker for the conversion of iRBD patients to parkinsonism or cognitive impairment is important for the clinical management and prognosis of both conditions. When applying our model to iRBD patients without an overt neurological condition (33 iRBD-only samples), we classified six individuals as pre-DLB patients while the rest would classify as pre-PD patients. Remarkably, five of the putative pre-PD patients, were classified as such with a sensitivity of 100%. Hence, we will examine the potential future clinical utility of our model by following closely the clinical evolution of these five individuals in the ensuing years at our institution.

We have found that the putative prediction to distinguish iRBD patients that will manifest parkinsonism or dementia was mainly based on the changes in two inversely related parameters: the mean size of HDL particles (*HDL-Z*) and the number of small HDL particles (*small HDL-P*). The parameter *HDL-Z*, which measures HDL particle profile and its heterogeneity, is inversely associated with cardiovascular risk^38^. Nevertheless, these differences are not statistically significant in a univariate analysis and there are no studies to confirm or deny the differences in cardiovascular risk between iRBD patients that will initially manifest parkinsonism or dementia, which should be further tested in future validation studies in independent iRBD cohorts.

This study has some limitations. First, the metabolic profiling was done in serum samples from non-fasting individuals, which adds variability and represents a confounding factor that could modify the results. Still, we have used stringent statistical cut-off values to avoid false-positive and -negative results. Also, one would expect that the putative alterations would be present in all groups under study since all samples were taken in non-fasting conditions, as well as the fact that variability between individuals is greater than the one expected by fasting^15^. The variation of postprandial status is mainly in the *small VLDL* parameter^15^, which is not used in our models. Second, it would have been optimal to analyze a second prospective sample from control individuals as done for the paired sample analysis for DLB and PD to rule out the contribution of ageing to the observed differences. Yet, age is an inherent factor associated to neurodegenerative diseases. Third, the relative low number of samples per group limits the reliability of the models presented. Hence, monitoring the iRBD-only individuals for the next 5 years is necessary to confirm the reliability of the presented model with a clinical diagnosis. Finally, due to sample availability, some groups under study (i.e., control vs. post-DLB) showed different gender distributions. Still, the models here described correspond to comparisons without any statistically significant gender bias.

Strengths of our study include (1) the analyses of 101 samples from iRBD patients, which can be considered a large number in the iRBD field, (2) that the iRBD diagnosis was confirmed by video-polysomnography in all cases, (3) that some samples were obtained from the same patients longitudinally before and after conversion to LTS, and (4) that machine learning techniques were used to build predictive models rather than using simple statistical comparisons between groups.

In conclusion, this study provides evidence of altered lipoprotein and protein glycosylation profiles in iRBD patients. Our results support that metabolic alterations and inflammation are involved in iRBD pathophysiology, and suggest biological differences underlying the progression of LTS in iRBD patients. We found that the baseline profiling of plasmatic lipoproteins and protein glycosylation in serum samples could potentially distinguish between iRBD patients that will eventually evolve to an LTS with either an initial development of parkinsonism or dementia. This information in combination with other clinical (e.g., smell tests showing hyposmia) and neuroimaging (e.g., DAT-SPECT showing dopamine transporter deficit in the striatum) markers could allow to better identify subjects with a high risk for short-term conversion to an overt LTS. Although this study requires an independent validation with larger and longitudinal cohorts to confirm the putative predictive value of the models here described, it provides an approach to a more accurate classification of iRBD patients in subtypes or variants with similar prognosis and underlying biology. If confirmed, this approach will help the design of future intervention studies to target more homogeneous subsets of LTS at early stages.

## Methods

### Participants selection

Polysomnographic-confirmed iRBD patients were recruited prospectively between 1996 and 2015 at the center for sleep disorders of the Neurology Service from the Hospital Clínic of Barcelona, Spain^39^. Patients and controls were Caucasians of Spanish origin. In all patients, iRBD was diagnosed by increased electromyography activity in the four limbs and in the chin associated with abnormal behaviors during REM sleep (e.g. punching, kicking, shouting). After iRBD diagnosis, patients were periodically followed at least every 6–12 months by a neurologist. Blood sample donation occurred during these routine visits in non-fasting conditions. If PD or DLB were suspected in the routine visits, iRBD patients were then examined by movement disorders or dementia expert neurologists to confirm the diagnosis. Diagnosis criteria were those accepted for PD^1^ and DLB^2^. When iRBD patients converted to PD or DLB, they were asked to donate a blood sample, either again if already participating in the study (paired samples) or for the first time if newly recruited.

Samples from a total of 82 iRBD patients were obtained. From these, the following age- and sex-matched groups were distinguished: (i) 33 individuals that remained disease-free at the end of the study (July 2019) (iRBD-only), (ii) 33 iRBD individuals that had no overt neurological disease at sample collection but later on converted to LTS (pre-LTS), among which there were (ii-a) 15 iRBD individuals that later on converted to DLB (pre-DLB), and (ii-b) 18 iRBD individuals that later on converted to PD (pre-PD), and (iii) 35 iRBD individuals that at sample collection had already converted to an LTS (post-LTS), among which there were (iii-a) 20 individuals that had already converted to DLB (post-DLB), and (iii-b) 15 individuals that had already converted to PD (post-PD). From the 33 iRBD patients who had no overt neurological disease at sample collection (ii), 19 converted later (i.e., 12 DLB and 7 PD) and a second sample was then collected and included within the 35 samples from the iRBD group with a diagnosed LTS (iii). Thus, from these 19 patients paired samples were available before and after the LTS diagnosis. We also included 29 samples from sex- and age-matched healthy controls without evidence of neurological or sleep disorders, which were selected from a database of healthy controls made of non-consanguineous attendants and volunteers who donate samples for research studies at our institution. All samples were processed and stored by the same person to avoid differences that could affect the results.

Demographic data are presented in Table 1, as well as relevant comorbidities (i.e., obesity, tobacco use, dyslipidemia, diabetes mellitus (type 2), and arterial hypertension) according to clinical records. Information about the medication taken by the participants and relevant to the outcome of the study (e.g., statins, antihypertensive drugs, diabetes medication) was sparse and not consistently reported for all cases, which precluded the possibility of including such information in our study.

### Standard protocol approvals, registrations, and patient consents

The ethical committee at the Hospital Clinic de Barcelona, Spain, approved the study and all participants gave written informed consent. Samples were registered at the biobank of IDIBAPS (S080327-01NL). Usage of the blood samples for research into disease biomarkers was approved by the Hospital Clínic Research Ethics Committee (HCB/2014/1065) and the Vall d’Hebron Hospital Research Ethics Committee (PR(AG)370/2014).

### Serum isolation

Five to 10 ml of blood were collected in tubes without anticoagulant (BD Vacutainer; Becton Dickinson, Franklin Lakes, NJ), preserved for 30 min at room temperature, and centrifuged at 1500 × *g* for 10 min at 4 °C. Serum volumes of 2 ml were removed from supernatant, aliquoted in polypropylene CryoTubes (Greiner Bio-One, Monroe, NC), flash frozen, and stored at −80 °C.

### 2D diffusion-ordered ^1^H NMR spectroscopy measurements

The lipoprotein profile and the presence of glycosylated proteins were measured in the 130 serum samples at the same time using the Liposcale® and the Glycoscale tests (Biosfer Teslab, Reus, Spain), respectively. The Liposcale® test is a CE marked and previously reported method based on 2D diffusion-ordered ^1^H NMR spectroscopy to estimate the size (-Z) of the three main types of lipoproteins (very low-density lipoprotein (VLDL), low-density lipoprotein (LDL), and high-density lipoprotein (HDL)), and the concentration (-P) of particles of three subtypes (large, medium, and small sized particles) of the main types of lipoproteins, as well as the lipid content [cholesterol (-C) and triglycerides (-TG)] of the three main classes, together with intermediate-density lipoproteins (IDL)^40^. The glycoscale uses the same technique to determine the presence of glycosylated proteins in serum, which is an indicator of systemic inflammatory processes^20^. More specifically, it determines *N*-acetlyglucosamine and *N*-acetylgalactosamine bound to protein (glycA), *N*-acetylneuraminic acid bound to protein (glycB), and any of the three acetyl groups not bound to protein (glycF). From these three peaks, it distinguishes their area (associated with concentration) and the ratio hight/width (describing the peak shape).

### Data analyses

#### Unpaired analysis

Differences between groups were assessed for those informative comparisons in terms of disease pathophysiology and with a diagnostic or prognostic value. These include control vs. pre-LTS, pre-DLB, pre-PD, post-LTS, post-DLB and post-PD, pre-LTS vs. post-LTS, pre-DLB vs. pre-PD, post-DLB vs. post-PD, pre-DLB vs. post-DLB, and pre-PD vs. post-PD. Since individuals in the iRBD-only group are a heterogeneous mixture of possible pre-LTS and individuals who might not convert during their lifetime, we excluded comparisons between them and any other group for being non-informative. The differences between comparisons were determined by a Wilcoxon signed-rank test combined with the Benjamini–Hochberg procedure to correct for false discovery rate (FDR) for any given comparison (cut-off at adjusted *p* value < 0.05). Results are expressed as median ± interquartile range.

#### Paired analysis

Paired samples (pre-LTS vs. post-LTS, pre-DLB vs. post-DLB, and pre-PD vs. post-PD, from the same patients) were (re)analyzed independently using the paired and non-parametric test Wilcoxon matched-pairs signed-rank test. Correction for multiple comparisons was done by controlling the FDR with the Benjamini–Hochberg procedure (cut-off at adjusted *p* value < 0.05).

#### Model building

Variables that allowed distinguishing between all groups were determined by non-parametric Kruskal–Wallis test correcting for multiple comparisons by controlling the FDR with the Benjamini–Hochberg procedure. Those variables significant with an FDR-corrected *p* value < 0.05 were used to determine which groups were different by non-parametric multiple comparisons of the mean rank of each group with the mean rank of every other group correcting for FDR by the Benjamini–Hochberg procedure. This allowed the selection of comparisons with differences (FDR-corrected *p* value < 0.05). Next, elastic net (R function *cv.glmnet*) was used on all variables from the chosen comparisons for variable selection and model building^41^. The elastic net mixing parameter was tested from 0.1 to 0.9, at intervals of 0.05, and a 10-fold cross-validation was performed. The model with the lowest mean cross-validated error was kept, and a receiver operating characteristics curve was made. Internal validation of the model was achieved by bootstrapping^42^, using the R function *vboot.glm*. Internal bootstrapping validation logistic model was done with 1000 bootstrap samples, and 10-fold cross-validation with 100 replicates. The AUC from each model was corrected by subtracting the optimism value obtained by bootstrapping, and only those models with a corrected AUC > 0.75 were kept.

Data analyses were performed in R^43^ and GraphPad Prism version 8.0.1 for Windows (GraphPad Software, La Jolla, California, USA, www.graphpad.com).

### Reporting summary

Further information on research design is available in the Nature Research Reporting Summary linked to this article.

## Supplementary information

Supplementary Table 1

REPORTING SUMMARY

## Data Availability

The data that support the findings of this study are available from the corresponding authors upon reasonable request.
