# Supplementary material for: Serum metabolic biomarkers for synucleinopathy conversion in isolated REM sleep behavior disorder
Source: NPJ Parkinsons Dis. 2021 May 13;7:40. doi: 10.1038/s41531-021-00184-9 (PMC8119407; doi:10.1038/s41531-021-00184-9)
Supplement: Supplementary file 1 — Supplementary Table 1 [file 41531_2021_184_MOESM1_ESM.pdf]

Supplementary Table 1. Lipoprotein and glycosylation profiling parameters quantified in serum samples

	Control	iRBD-only	Pre-LTS	Pre-LTS	Pre-DLB	Pre-DLB	Pre-PD	Pre-PD	Post-LTS	Post-LTS	Post-DLB	Post-DLB	Post-PD	Post-PD
			All	Paired	All	Paired	All	Paired	All	Paired	All	Paired	All	Paired
Area Glyc-A (µmol/L)	1019 ± 182	920 ± 236	926 ± 216	919 ± 116	960 ± 220	949 ± 142	898 ± 206	880 ± 58	911 ± 184	920 ± 199	909 ± 178	925 ± 148	956 ± 179	841 ± 207
Area Glyc-B (µmol/L)	431 ± 120	416 ± 47	378 ± 88	368 ± 69	**335 ± 67**[1]	333 ± 56	396 ± 85	392 ± 19	390 ± 104	**416 ± 85**[2]	381 ± 95	402 ± 72	395 ± 92	450 ± 51
Area Glyc-F (µmol/L)	366 ± 147	349 ± 97	346 ± 135	345 ± 77	377 ± 92	382 ± 88	329 ± 117	328 ± 74	330 ± 65	341 ± 68	328 ± 65	346 ± 60	345 ± 60	321 ± 67
H/W Glyc-A (AA)	22.18 ± 3.77	21.71 ± 5.78	21.54 ± 3.75	21.34 ± 2.08	21.83 ± 2.62	21.69 ± 2.69	21.26 ± 5.49	21.17 ± 1.13	20.83 ± 3.65	21.36 ± 3.51	20.90 ± 4.21	22.08 ± 3.41	20.83 ± 2.82	19.89 ± 1.99
H/W Glyc-B (AA)	5.56 ± 1.48	5.3 ± 1.31	5.64 ± 0.91	5.59 ± 0.62	5.70 ± 0.71	5.65 ± 0.61	5.52 ± 1.02	5.56 ± 0.76	5.44 ± 0.88	5.31 ± 1.04	5.52 ± 1.33	5.42 ± 1.08	5.40 ± 0.82	5.23 ± 0.45
HDL-C (mg/dL)	62.7 ± 23.25	57.19 ± 16.07	58.26 ± 21.05	60.22 ± 20.07	54.15 ± 22.03	58.55 ± 19.37	58.65 ± 20.47	60.22 ± 18.46	53.27 ± 18.55	55.76 ± 17.71	56.19 ± 19.68	55.90 ± 14.71	50.54 ± 14.47	55.42 ± 23.24
HDL-L (µmol/L)	0.34 ± 0.01	0.28 ± 0.09	0.30 ± 0.07	0.30 ± 0.04	0.32 ± 0.07	0.32 ± 0.06	0.30 ± 0.09	0.30 ± 0.05	0.29 ± 0.07	0.27 ± 0.08	0.29 ± 0.08	0.28 ± 0.06	0.26 ± 0.08	0.23 ± 0.11
HDL-M (µmol/L)	11.14 ± 4.86	9.21 ± 2.58	10.42 ± 1.93	10.51 ± 1.30	10.42 ± 1.94	10.61 ± 1.11	10.39 ± 2.36	10.50 ± 1.48	9.87 ± 2.21	9.94 ± 2.21	10.11 ± 2.	10.11 ± 1.68	9.47 ± 2.58	8.95 ± 3.95
HDL-S (µmol/L)	20.57 ± 7.77	20.78 ± 4.35	19.66 ± 5.22	20.16 ± 4.51	18.39 ± 5.74	20.17 ± 3.81	19.91 ± 5.66	20.16 ± 6.54	17.16 ± 6.22	18.46 ± 4.19	17.35 ± 3.71	17.72 ± 3.57	17.04 ± 6.74	19.94 ± 6.42
HDL-TG (mg/dL)	18.03 ± 7.23	14.81 ± 5.12	15.16 ± 7.11	16.49 ± 4.39	17.37 ± 7.38	18.74 ± 6.35	14.72 ± 5.97	14.97 ± 2.68	15.02 ± 5.17	14.42 ± 6.10	14.93 ± 6.35	14.55 ± 7.07	16.25 ± 6.28	14.42 ± 4.79
HDL-Z (nm)	8.27 ± 0.08	8.22 ± 0.11	8.25 ± 0.09	8.25 ± 0.04	8.25 ± 0.08	8.26 ± 0.04	8.24 ± 0.11	8.24 ± 0.06	8.28 ± 0.07	8.27 ± 0.07	8.28 ± 0.07	8.28 ± 0.07	8.28 ± 0.09	8.27 ± 0.06
IDL-C (mg/dL)	10.42 ± 6.45	9.18 ± 5.76	9.37 ± 6.99	9.37 ± 5.81	12.33 ± 7.4	12.65 ± 7.04	8.73 ± 5.03	8.22 ± 2.09	9.27 ± 3.32	8.94 ± 3.69	9.25 ± 4.2	9.17 ± 4.83	9.91 ± 4.56	7.02 ± 2.93
IDL-TG (mg/dL)	12.68 ± 5.09	11.17 ± 4.08	11.59 ± 6.14	11.51 ± 3.20	12.3 ± 5.32	12.22 ± 4.71	10.78 ± 4.69	10.67 ± 1.52	10.99 ± 3.42	11.00 ± 3.58	10.93 ± 3.82	11.31 ± 4.06	10.99 ± 4.33	9.37 ± 2.51
LDL-C (mg/dL)	105 ± 43	112 ± 28	109 ± 43	119 ± 38	119 ± 41	119 ± 42	102 ± 42	106 ± 33	108 ± 36	107 ± 39	108 ± 33	108 ± 31	108 ± 46	88 ± 61
LDL-L (nmol/L)	185 ± 55	169 ± 48	180 ± 73	184 ± 60	191 ± 57	197 ± 52	160 ± 69	161 ± 45	182 ± 66	178 ± 55	183 ± 48	181 ± 51	175 ± 72	152 ± 95
LDL-M (nmol/L)	391 ± 186	322 ± 158	376 ± 180	418 ± 142	418 ± 161	426 ± 141	343 ± 166	367 ± 137	338 ± 162	**305 ± 134**[2]	335 ± 144	314 ± 92	344 ± 179	241 ± 211

LDL-S (nmol/L)	601 ± 164	639 ± 114	595 ± 201	596 ± 197	598 ± 235	597 ± 243	585 ± 171	594 ± 107	612 ± 153	612 ± 199	612 ± 151	618 ± 132	590 ± 181	522 ± 247
LDL-TG (mg/dL)	20.27 ± 7.02	16.91 ± 7.24	18.96 ± 8.98	19.79 ± 10.57	22.07 ± 11.38	24.18 ± 10.33	17.55 ± 5.99	18.84 ± 4.67	17.90 ± 5.64	**16.68 ± 6.18**[2]	17.29 ± 5.98	17.40 ± 6.36	18.07 ± 7.22	15.75 ± 7.52
LDL-Z (nm)	21.06 ± 0.24	20.9 ± 0.42	21.10 ± 0.33	21.10 ± 0.16	21.16 ± 0.22	21.17 ± 0.17	21.04 ± 0.35	21.07 ± 0.10	21.04 ± 0.37	20.93 ± 0.22	21.03 ± 0.36	20.98 ± 0.23	21.05 ± 0.37	20.91 ± 0.20
VLDL-C (mg/dL)	25.12 ± 14.93	22.61 ± 16.42	20.14 ± 20.53	20.14 ± 15.53	24.9 ± 23.31	24.02 ± 17.96	14.74 ± 18.67	15.84 ± 8.87	19.16 ± 14.12	19.16 ± 13.92	19.08 ± 11.24	20.13 ± 10.02	20.51 ± 17.25	15.12 ± 16.49
VLDL-L (nmol/L)	1.61 ± 0.82	1.5 ± 0.76	1.11 ± 0.85	1.10 ± 0.73	1.40 ± 1.12	1.39 ± 0.64	0.95 ± 0.86	0.89 ± 0.27	1.16 ± 0.63	1.14 ± 0.70	1.14 ± 0.71	1.14 ± 0.73	1.33 ± 0.61	1.26 ± 0.51
VLDL-M (nmol/L)	9.22 ± 6.86	7.55 ± 6.17	5.37 ± 5.72	5.63 ± 4.48	6.77 ± 4.75	7.34 ± 3.85	4.11 ± 5.31	4.36 ± 1.70	5.92 ± 3.68	5.88 ± 4.36	5.74 ± 4.21	5.74 ± 3.35	7.04 ± 3.8	6.44 ± 4.63
VLDL-S (nmol/L)	56.84 ± 30.91	54.28 ± 32.06	36.99 ± 40.47	36.99 ± 32.13	58.49 ± 41.37	50.93 ± 32.36	31.35 ± 33.85	30.38 ± 12.35	40.71 ± 27.81	40.63 ± 29.01	38.85 ± 28.32	43.58 ± 28.30	44.71 ± 29.28	37.14 ± 26.01
VLDL-TG (mg/dL)	85.62 ± 57.12	86.94 ± 54.15	52.24 ± 58.46	52.54 ± 43.22	78.09 ± 58.86	71.48 ± 44.77	45.61 ± 50.84	45.28 ± 14.08	63.01 ± 40.41	63.01 ± 46.06	56.49 ± 43.46	62.19 ± 42.72	63.53 ± 39.87	63.01 ± 39.60
VLDL-Z (nm)	42.23 ± 0.19	42.17 ± 0.18	42.15 ± 0.18	42.19 ± 0.16	42.15 ± 0.25	42.17 ± 0.18	42.15 ± 0.14	42.20 ± 0.13	42.18 ± 0.16	42.20 ± 0.14	42.17 ± 0.2	42.19 ± 0.14	42.19 ± 0.16	42.22 ± 0.18
LDL-P/HDL-P	34.72 ± 13.43	37.34 ± 11.9	39.97 ± 15.9	39.68 ± 11.65	46.51 ± 23.09	40.03 ± 19.87	37.88 ± 11.78	39.09 ± 5.80	38.79 ± 10.22	37.46 ± 8.21	38.72 ± 6.56	38.13 ± 7.70	39.71 ± 13.43	35.51 ± 8.97
Total-P/HDL-P	36.67 ± 13.25	38.56 ± 12.17	41.63 ± 18.06	39.15 ± 9.37	50.2 ± 24.81	40.75 ± 10.9	40.75 ± 13.03	37.53 ± 7.44	40.76 ± 10.58	40.95 ± 13.43	40.75 ± 8.11	42.52 ± 21.27	42.23 ± 12.85	40.69 ± 6.93

Quantifications are shown for the whole group, including both paired and unpaired samples (All), and only for paired samples (Paired). Data are shown as median ± interquartile range. AA, arbitrary units; DLB, dementia with Lewy bodies; GlycA, N-acetlyglucosamine and N-acetylgalactosamine bound to protein; GlycB, N-acetylneuraminic acid bound to protein; GlycF, N-acetlyglucosamine, N-acetylgalactosamine or N-acetylneuraminic acid not bound to protein; HDL, high-density lipoprotein; IDL, intermediate-density lipoprotein; iRBD, isolated rapid eye movement (REM) sleep disorder; LDL, low-density lipoprotein; LTS, Lewy-type synucleinopathy; PD, Parkinson's disease; Pre, samples obtained before the onset of the overt neurodegenerative disease; Post, samples obtained after the onset of the overt neurodegenerative disease; VLDL, very low-density lipoprotein; H/W, height/width; C, cholesterol; L, large particles; M, medium particles; S, small particles; Z, particle diameter; TG, triglycerides. Age and time differences from the whole group (All) and the paired samples (Paired) were assessed by Wilcoxon signed-rank test or the paired and non-parametric test Wilcoxon matched pairs signed rank test, respectively, combined with the Benjamini–Hochberg procedure to correct for false discovery rate. [1]Significantly different from control; [2]Significantly different from pre-LTS.
